# Exopolysaccharide production by seven basidiomycetous fungi and their antioxidant and immunomodulatory activities against *Salmonella* infection

**DOI:** 10.3389/fcimb.2025.1610403

**Published:** 2025-06-16

**Authors:** Thanawut Chotmanee, Nakarin Suwannarach, Jaturong Kumla, Rewat Phongphisutthinant, Supakit Chaipoot, Pairote Wiriyacharee, Arishabhas Tantibhadrasapa, Songbo Li, Parameth Thiennimitr, Saisamorn Lumyong

**Affiliations:** ^1^ Faculty of Science, Chiang Mai University, Chiang Mai, Thailand; ^2^ Department of Biology, Faculty of Science, Chiang Mai University, Chiang Mai, Thailand; ^3^ Center of Excellence in Microbial Diversity and Sustainable Utilization, Chiang Mai University, Chiang Mai, Thailand; ^4^ Office of Research Administration, Chiang Mai University, Chiang Mai, Thailand; ^5^ Multidisciplinary Research Institute, Chiang Mai University, Chiang Mai, Thailand; ^6^ Department of Microbiology, Faculty of Medicine, Chiang Mai University, Chiang Mai, Thailand; ^7^ Academy of Science, The Royal Society of Thailand, Bangkok, Thailand

**Keywords:** antioxidant potential, fungal exopolysaccharide, immunomodulatory, liquid cultivation, mushroom mycelium, MDR *Salmonella*

## Abstract

The discovering new fungal strains, optimal production, and understanding the fundamental aspects of exopolysaccharides (EPs) are important to utilize them in an industrial, medical, and biotechnological perspective. In this study, the optimal conditions for EP production from seven basidiomycetous fungal strains were investigated. The results indicated that six fungal species, *Schizophyllum commune*, *Ganoderma fornicatum*, *G*. *williamsianum*, *Earliella scabrosa*, *Favolus tenuiculus*, and *Pycnoporus sanguineus*, produced the highest EP yield in potato dextrose broth. The highest yield of EPs produced by *Lentinus sajor*-*caju* was obtained in mushroom complete medium broth. It was found that a pH value between 6 and 8 in the liquid culture media promoted EP production. The highest EP yield was obtained for 10 to 14 days which depends on fungal strain. Interestingly, this present study revealed the first report of EP production from *G. fornicatum*, *G*. *williamsianum*, *E. scabrosa*, *F. tenuiculus*, and *P. sanguineus*, including the genera *Earliella* and *Favolus*. The obtained crude EPs showed water solubilization ability. The Fourier-transform infrared spectroscopy spectra exhibited typical carbohydrate patterns in all crude EPs. Monosaccharide composition analysis revealed that the crude EPs were primarily composed of glucose, followed by fructose, allose, and allulose, with variations depending on the fungal strain. Additionally, crude EPs demonstrated positive antioxidant potential. Finally, we determined the anti-*Salmonella* and immunomodulatory effects of crude EPs from *S. commune*, *G. fornicatum*, and *L. sajor-caju* due to their high EP yield. Pretreatment of mouse macrophages with these fungal EPs enhanced the phagocytic killing activity of *Salmonella*-infected macrophages. Upregulations of pro-inflammatory cytokine expression in macrophages were detected in the fungal EPs-treated groups. Our study reported the optimizing conditions for EP production from several strains of Basidiomycetous fungi and their potential as an alternative to antibiotics for multidrug-resistant *Salmonella* infection.

## 1 Introduction

Fungi are a diverse group of microorganisms that can produce a wide range of biologically active compounds, including extracellular polysaccharides or exopolysaccharides (EPs). EPs are complex carbohydrates that are secreted by fungi into their environment, forming a protective matrix around the cells (Valasques et al., 2020). Mushrooms, which mostly belong to the phylum Basidiomycota, are fungal fruiting bodies that are well known for their nutritional and medicinal properties (Miles and Chang, 2004). Notably, mushrooms contain diverse polysaccharides, including schizophyllan, ganoderan, grifolan, pleuran, and lentinan, which are recognized as valuable biomacromolecules (Mahapatra and Banerjee, 2013; Nguyen et al., 2024; Prathumpai et al., 2025). However, cultivating mushrooms as a source of polysaccharides requires a large volume of substrate and space, several months, and the quality of the final product can be relatively low depending on the substrate and growing conditions (Gong et al., 2020; Leong et al., 2021). As an alternative approach, liquid and submerged cultivation of mushroom mycelia has emerged as a promising method for producing polysaccharides (Mao et al., 2014). Some mushroom mycelia can produce and secrete EPs during liquid and submerged cultivation, offering an attractive means of obtaining high-value EPs. EPs provide several advantages over intracellular and cell wall polysaccharides, including massive production, easy isolation, and purification in a short time (Yan et al., 2010; Liu et al., 2024). Remarkably, the production of EPs from fungal mycelia is influenced by a variety of factors, including genetic factors (such as species and strains), physical or environmental conditions (such as pH, temperature, and aeration) during cultivation, and nutrient availability (including carbon and nitrogen sources, as well as trace minerals) in the culture medium. Several studies have focused on the optimization of culture conditions for the production of fungal EPs, with the aim of improving yields and reducing production costs (Kim et al., 2006, 2010; Meng et al., 2010; Patel et al., 2014; Tabibzadeh et al., 2022; Thai and Keawsompong, 2019). Understanding these factors is essential for producing and utilizing fungal EPs to their full potential in a variety of applications. This knowledge could lead to the development of new, sustainable, and cost-effective methods for EP production.

Exopolysaccharides (EPs) have various applications in industries, pharmaceuticals, medicines, and foods. The production of EPs from mushroom mycelia through liquid and submerged cultures has been widely studied, including genera *Agaricus*, *Cordyceps*, *Ganoderma*, *Grifola*, *Lentinus*, *Pleurotus*, *Schizophyllum*, *Trametes*, *Tricholoma* and others (Mahapatra and Banerjee, 2013). Recent research has unveiled the link between mushroom EPs and human health. A number of EPs from the culture filtrate of mushroom mycelia have been exhibited with a range of interesting biological and pharmacological activities, such as anti-inflammatory, antitumor, antimicrobial, immunomodulatory, and antioxidant activities (Kheni and Vyas, 2017; Muszyńska et al., 2018; Ghosh et al., 2021). However, research on EP production in Thailand remains limited. Therefore, this study aimed to screen and select basidiomycetous fungal strains for EP production. The optimal conditions (type of liquid culture medium, pH, and cultivation period) for EP production in each fungal strain were investigated. Subsequently, the antioxidant activities, major structural characteristics, and chemical compositions of the obtained crude EPs were analyzed. The immunomodulatory effects of the selected crude fungal EPs were evaluated by assessing the mRNA expression of pro-inflammatory and chemo-attractant cytokine genes in RAW264.7 macrophage cells, as well as the macrophage intracellular survival of *Salmonella enterica* serovar Typhimurium (STM). The information obtained from this study can be valuable for enhancing EP production and developing crude fungal EPs for medicinal and other applications.

## 2 Materials and methods

### 2.1 Source of fungal strains

A total of seven fungal strains (including *Earliella scabrosa* NK0461, *Favolus tenuiculus* NK0550, *Ganoderma fornicatum* NK0524, *Ganoderma williamsianum* NK0540, *Lentinus sajor-caju* NK0427, *Pycnoporus sanguineus* NK0189, and *Schizophyllum commune* CMU-01) in this study were obtained from the culture collection of the Research Center of Microbial Diversity and Sustainable Utilization, Faculty of Science, Chiang Mai University, Thailand. The fungal mycelia were cultured on potato dextrose agar (PDA; Conda, Madrid, Spain) and kept in incubator at 30°C for 7 days.

### 2.2 EP production

The ability of each fungal strain to produce EPs was investigated in liquid culture. The cultivation was carried out in a 125-mL Erlenmeyer flask containing 40 mL of mushroom complete medium broth (MCMB; containing glucose 20 g/L, peptone 2 g/L, yeast extract 2 g/L, K_2_HPO_4–_1 g/L, MgSO_4_•7H_2_O 0.5 g/L and KH_2_PO_4_ 0.46 g/L) with initial pH value at 6.0 (Jeong et al., 2009). Three mycelial plugs (5 mm in diameter) of each fungal strain obtained from a colony growing on PDA at 30°C for 7 days were inoculated into MCMB. The inoculated flasks were incubated at room temperature (28 ± 2°C) on a reciprocal shaker (NR-10 Bioshaker^®^, Japan) at 110 rpm. After 7 days, the fungal mycelia were filtered through a 25-µm membrane filter (Miracloth, Merck Millipore, USA), and the culture supernatant was collected for crude EP extraction. The fungal strains positive for EP production were selected and used for further experiments.

### 2.3 Precipitation of EPs

The fungal EPs were precipitated using 75% ethanol and the fungal culture supernatant in ratios of 2:1 (v/v), sealed with parafilm, and kept at 4°C according to the method described by Long et al. (2021) and Yurnaliza et al. (2021). After 24 h, EPs were collected by centrifuged at 10,000×g for 15 minutes and washed with 95% ethanol three times. Then, the obtained crude EPs were dissolved in deionized (DI) water and freeze-dried by a lyophilizer at -50°C (FreeZone 2.5 Liter Benchtop Freeze Dryer, Labconco) until completely dry. The crude fungal EPs were weighed and stored at -20°C until further experiments.

### 2.4 Optimization of culture conditions for enhanced EP production

The EP production of each fungal strain was investigated in 250-mL Erlenmeyer flasks containing 80 mL of the tested liquid culture under various conditions (type of liquid culture media, initial pH values, and incubation time). Five mycelial plugs of each fungal strain were inoculated into each flask. The inoculated flasks were incubated at 28 ± 2°C on a reciprocal shaker at 110 rpm. The EPs were precipitated and collected using the method described above.

#### 2.4.1 Type of liquid culture media

Three different types of liquid culture media including MCMB, potato dextrose broth (PDB; Conda, Madrid, Spain) and yeast-malt extract broth (YMB; glucose 10 g/L, peptone 5 g/L, malt extract 3 g/L and yeast extract 3 g/L) with an initial pH level of 6.0 were used in this experiment. Mycelial plugs of each fungal strain were inoculated into each flask and cultivated on a shaker for two weeks. The EPs were precipitated and collected. The culture liquid medium of each fungal strain that provided the maximum EP yield was selected and used for further experiments.

#### 2.4.2 Initial pH values of optimal liquid media

Pure cultures of each fungal strain were inoculated into the selected liquid culture medium with six different initial pH values (4.0, 5.0, 6.0, 7.0, 8.0, and 9.0). The cultivation was performed for two weeks, and the EPs were precipitated and collected. The initial pH value of the liquid medium that resulted in the highest EP yield for each fungal isolate was selected for further experiments.

#### 2.4.3 Incubation time

The influence of the incubation time on EP production for each fungal strain was evaluated in the optimal liquid culture medium with an optimal initial pH value. After inoculation with pure culture, flasks were placed on a reciprocal shaker at 28 ± 2°C. The EP production yield was examined after 6, 8, 10, 12, 14, and 16 days of incubation.

### 2.5 Fourier transform-infrared spectroscopy (FTIR) analysis

FT-IR analysis was performed at Science and Technology Service Center, Faculty of Science, Chiang Mai University, Thailand. The FT-IR spectrum was used to elucidate the structural analysis of the crude EPs as described by Tabibzadeh et al. (2022) with some modification. The samples were prepared by mixing 2 mg of grinded EPs with 200 mg of spectroscopic-grade potassium bromide (KBr) powder, which was pulverized and compressed into 1 mm pellets for FT-IR determination. The absorption was measured and analyzed using a FT-IR spectrophotometer (Thermo Fisher Scientific, USA) in the wavenumber region of 400 to 4,000 cm^-1^ for detecting the presence of major structural and functional groups of EPs along with their substitutes. Schizophyllan was used as the standard for comparison.

### 2.6 Chemical characterization for sugar compositions of seven fungal EPs

Monosaccharide compositions of crude fungal EPs were determined by high performance liquid chromatography (HPLC) according to the method of Du et al. (2017) and Long et al. (2021). The crude EPs were prepared by dissolving 1 g of them in 10 mL of distilled water. The solution was then digested using 1M HCl, with the pH adjusted to 2, followed by heating to 97°C and maintaining it for 5 min. Subsequently, the pH was adjusted to 7 using 1M NaOH, and the mixture was centrifuged at 8,000 rpm at 25°C for 10 min. The clear supernatant was subjected to analysis for sugar compositions using a Shimadzu-brand high-performance liquid chromatography machine with an isocratic technique, utilizing a Shodex HILICpak VG-50 4E column and a refractive index (RI) detector. The column temperature was maintained at 50°C. For the analysis, the mobile phase comprised a mixture of acetonitrile, methanol, and water in a ratio of 85:10:5 (solvent A), HPLC-grade water (solvent B), and 100% acetonitrile (solvent C), with a flow rate set at 0.6 mL/min. The injection volume was 10 μL. The presence of monosaccharides was identified by comparing the retention time of allose, allulose, fructose, mannose, glucose, rhamnose, and xylose standards. The amount of each monosaccharide was quantified with the calibration curve constructed with each standard.

### 2.7 Antioxidant assays

Evaluation of the antioxidant activities of EPs was determined in a 96-well microplate by two methods, including 2,2’-azino-bis (3-ethylbenzthiazoline-6-sulphonic acid) diammonium salt (ABTS) and 1,1-diphenyl-2-picrylhydrazyl (DPPH) radical scavenging assays. For all assays, the EP samples were dissolved in deionized water. Gallic acid was used as a reference compound (positive control) both ABTS and DPPH assay. All samples were evaluated in triplicate. The ABTS and DPPH radical scavenging activities were expressed as the IC_50_ value (half maximal inhibitory concentration) in mg/mL of the EP sample required to inhibit 50% of ABTS or DPPH radical scavenging. The IC_50_ values were obtained by interpolation from linear regression analysis. A lower IC_50_ value corresponds to higher antioxidant activity.

#### 2.7.1 ABTS radical scavenging activity

The ABTS radical scavenging activities of EPs were performed spectrophotometrically by decolorization assay according to the methods of Prajapati et al. (2023) and Wangsawat et al. (2021) with slight modifications. An ABTS radical cation solution was prepared by reacting equal volumes (500 µl) of 7 mM ABTS solution with 2.45 mM potassium persulfate (K_2_S_2_O_8_) solution. Then, the mixture containing free radicals was kept in the dark for 16 hours at room temperature to yield a dark-colored solution before use. After that, the stock solution was diluted with deionized water to give an absorbance of 0.70 ± 0.02 at 734 nm. The ABTS working solution (195 μL) was added to 5 μL of the EP solution with different concentrations. A mixture of deionized water and ABTS solution was used as negative control. After incubation for 10 minutes in the dark, the final absorbance of the reaction was measured at 734 nm with a microplate reader spectrophotometer (Spectra MR, Dynex Technologies, Virginia). The inhibition of the ABTS radical was calculated as IC_50_ and expressed in mg/mL.

#### 2.7.2 DPPH radical scavenging activity

The DPPH radical scavenging activities of EPs were carried out by the methods described by Debnath et al. (2021) and Xu et al. (2015) with minor modifications. In brief, 50 µL of each EP sample at different EP concentrations in deionized water was mixed with DPPH radical solution in 95% ethanol (0.1 mM, 150 µL). The mixture was shaken vigorously are allowed to stand for 30 minutes in the dark. A mixture of deionized water and DPPH solution was used as negative control. Then, the absorbance of each mixture solution was spectrometrically measured at 517 nm using a microplate reader spectrophotometer (Spectra MR, Dynex Technologies, Virginia). The DPPH radical scavenging ability of EPs was calculated as the IC_50_ value and expressed in mg/mL.

### 2.8 Effect of crude fungal EPs on the cell viability of RAW264.7 cells

Murine macrophage cell lines (RAW264.7 cells) purchased from the American Type Culture Collection (ATCC^®^ TIB-71™) were used in this study. RAW264.7 cells were cultured in Dulbecco’s modified Eagle medium (DMEM; HyClone, United States) high glucose containing 4mM L-glutamine, 4.5 g/L glucose, and sodium pyruvate supplemented with 10% fetal bovine serum (FBS; Hyclone, United States) and 1% penicillin/streptomycin (Gibco, United States). The cells were changed to antibiotic‐free media before using in an assay. These cells were maintained in an incubator with 5% CO_2_-humidified air at 37°C. The cell culture media were changed every 2–3 days.

The crude EPs from *S. commune*, *G. fornicatum*, and *L. sajor-caju* were selected for this study based on their high production yields. To assess the cytotoxicity of fungal EPs on RAW264.7 cells, cell viability was determined using the MTT (3-[4,5-dimethylthiazol-2-yl]-2,5-diphenyl-tetrazolium bromide) assay following He et al. (2019) with slight modifications. Initially, 100 μL of RAW264.7 cells were seeded into a 96-well plate at a density of 1.0×10^4^ cells/well and incubated at 37°C with 5% CO_2_ for 24 hours to achieve stable growth. The cells were then cultured with antibiotic-free media for an additional 24 hours. Subsequently, the cells were treated with various final concentrations of crude fungal EPs in PBS solution at concentrations of 25, 50, 100, 200, 400, 500, and 1000 µg/mL. PBS was used as the solvent for EP dilution and for the EPs-untreated control group. After 24 hours of incubation, the treatments were discarded, and 300 µL of MTT solution (1 mg/mL in PBS) was added to each well. The plates were then incubated for an additional 3 hours at 37°C. Formazan crystals formed in viable cells were dissolved by adding 150 µL of DMSO/well, and the supernatant was gently shaken on a plate shaker for 10 minutes. Absorbance values (Abs) were determined with a reference wavelength of 490 nm using a microplate reader (BioTek Synergy H4 Hybrid Multi-Mode Microplate Reader, Marshall Scientific). Increased production of formazan directly indicates the presence of a higher number of metabolically active cells. The percentage of cell viability was calculated using the formula:


$$\text{Cell viability }(\%)=(\text{Ab}{\text{s}}_{\text{sample}}/\text{Ab}{\text{s}}_{\text{control}})\times 100$$

Where Abs _control_ represents the absorbance of the control reaction (without EP treatment), and Abs _sample_ represents the absorbance of the EP treatment. Cell viability was expressed as a percentage of the control, represented by EPs-untreated cells, which were set at 100%. Due to the known variability of the MTT assay, experiments were conducted with eight replicates to ensure statistical robustness.

### 2.9 Effect of fungal EPs on the macrophage intracellular survival of *Salmonella*

#### 2.9.1 Bacterial strain

*Salmonella enterica* serovar Typhimurium strain IR715 (ATCC 14028 derivative with nalidixic acid resistance; STM) was used. STM was grown at 37°C aerobically with shaking in Luria-Bertani (LB) broth (10 g/L tryptone, 5 g/L yeast extract, and 10 g/L NaCl; Difco, United States) for 16–18 hours. STM was sub-cultured for 3 hours in LB broth before treating RAW264.7 cells. Nalidixic acid (0.05 mg/mL; AppliChem, Germany) was used as a selective antibiotic for STM in liquid and solid media.

#### 2.9.2 Invasion assay

The effect of fungal EPs on murine macrophages to eliminate invasive STM was adapted and determined using the invasion assay previously described by Birhanu et al. (2018) with some modifications. Briefly, 500 µL of RAW264.7 cells suspended in a complete medium were seeded into 24-well plates at a density of 1.0×10^5^ cells/well and incubated at 37°C with 5% CO_2_ for 24 hours. The complete growth medium was then replaced with DMEM without FBS or antibiotics for an additional 24 hours to synchronize the cells. Subsequently, RAW 264.7 cells were pre-treated with different final concentrations of the three fungal EPs in PBS solution at 25, 50, 100, and 200 µg/mL for 24 hours, with PBS being used for the EPs-untreated control. Simultaneously, overnight cultures of STM were sub-cultured in LB with 0.3 M NaCl and incubated at 220 rpm, 37°C with 5% CO_2_ for 3 hours. Next, the cell lines were gently treated and infected by STM adding at the multiplicity of infection (MOI) of 5 at 37°C for 1 hour. The cell culture medium in each well was removed, and the bacterial cells were subsequently rinsed twice with Dulbecco’s phosphate-buffered saline (DPBS; Hyclone, Singapore) and treated with 500 μL of 100 μg/mL gentamicin sulfate (AppliChem, Germany) in DMEM, and incubated at 37°C for a further 90 minutes. The supernatant in each well was then removed, and the RAW264.7 cells were washed twice with DPBS before adding 500 µL of 1% Triton-X-100 (Thermo Fisher Scientific, United States) in PBS to lyse the cells. Subsequently, 500 µL of PBS was repeatedly used to collect the remaining bacterial cells in each well. The cell suspensions were ten-fold serially diluted and enumerated on LB agar supplemented with nalidixic acid to determine the viable numbers of macrophage intracellular STM. The experiment was performed with six replicates per group due to limitations in the available quantity of the EP sample.

#### 2.9.3 Pro-inflammatory and chemo-attractant cytokine genes expression of EPs-treated and STM-infected RAW264.7 cells

The expression of pro-inflammatory and chemo-attractant cytokine genes of EPs and STM-infected RAW264.7 cells was performed by reverse transcription and qualitative polymerase chain reaction (RT-qPCR), as previously described by Winter et al., 2009, and Buddhasiri et al., 2021, with some modifications. In brief, 2 mL of RAW264.7 cells suspended in complete medium were seeded into 6-well plates at a density of 1.2×10^6^ cells/well and incubated at 37°C with 5% CO_2_ for 24 hours. The complete medium was replaced 24 hours prior to the assay with DMEM without FBS and antibiotics. Subsequently, RAW264.7 cells were pre-treated with EP solution in PBS at two final concentrations of 50 and 200 µL/mL for 24 hours. PBS and STM suspended in PBS were used for the EPs-untreated and positive control groups, respectively. Following pretreatment, RAW264.7 cells were treated with STM at an MOI of 5 at 37°C for 1 hour. After two rounds of washing the cells with DPBS, they were lysed and collected by adding 1 mL of TRIzol^®^ reagent (Invitrogen Life Technologies, USA) into each well, then stored at -20°C prior to subsequent use.

The total RNA of RAW264.7 cells was extracted using TRIzol^®^ reagent following the manufacturer’s protocol recommendations. DNase treatment was carried out using a DNA clean-up and inactivation kit (Ambion, Life Technologies). RNA samples were quantified using a NanoDrop Spectrophotometer (Thermo Fisher Scientific, United States) and stored at -80°C until use. To ensure good quality and integrity, extracted RNA was evaluated by measuring the absorbance ratio of 260/280 (1.6-1.9) and running on a 1.5% agarose gel stained with SYBR Safe DNA Gel Stain (Thermo Fisher Scientific, USA). Subsequently, complementary DNA (cDNA) was synthesized by reverse transcription polymerase chain reaction (RT-PCR) using a RevertAid First Strand cDNA Synthesis Kit (Thermo Scientific). RT-PCR was performed by mixing extracted RNA (3 µg), Random Hexamer Primer, 10 mM dNTP mix, 5X-Reaction Buffer, RiboLock RNase Inhibitor, and RevertAid M-MuLV Reverse transcriptase (200µ/µL) in a total volume of 20 µL under the following conditions: incubation at 25°C for 5 minutes, 42°C for 60 minutes, with a termination step of 5 minutes at 70°C using a thermocycler (Labcycler, SensoQuest).

The quantitative PCR (qPCR) was performed using a reaction mixture consisting of 2X-SensiFAST™ SYBR^®^ Lo-ROX mix (Bioline), 10 µM of forward and reverse primers, 2 µL of 50 ng/µL cDNA template, and nuclease-free water to a final volume of 20 µL. The amplification program involved an initial polymerase activation at 95°C for 2 minutes, followed by 40 cycles of denaturation at 95°C for 5 seconds and an annealing step at 60°C for 30 seconds, as developed by Boonloh et al. (2015), using the ViiA7 Real‐Time PCR system (Applied Biosystems, United States). The threshold cycle (C_T_) values were exported to Microsoft Excel for further analysis. Melting curve analyses were utilized to confirm the positive sample specificity of each amplified PCR product. Positive and negative controls were included with each PCR run. The relative fold change of mRNA expressions was calculated using the comparative C_T_ (2^−ΔΔCT^) method (Schmittgen and Livak, 2008). The expression levels of pro-inflammatory cytokine genes, including *Il6*, *Mip2*, *Nos2*, and *Tnfα*, encoding interleukin (IL)‐6, macrophage inflammatory protein (MIP)‐2 or chemokine (C-X-C motif) ligand 2 (CXCL2), inducible nitric oxide synthase (iNOS), and tumor necrosis factor (TNF)‐*α*, were normalized with the stably expressed housekeeping gene (*Gapdh*). The primer pairs used for the qPCR gene expression in this study are presented in **Table 1**.

**Table 1:** Primer sequences of genes investigated by qPCR analysis.

Genes	Primer Sequences		References
*Gapdh*	Forward	5′-TGTAGACCATGTAGTTGAGGTCA-3′	Winter et al. (2010)
	Reverse	5′-AGGTCGGTGTGAACGGATTTG-3′	
*Nos2*	Forward	5′-CCAGCCTTGCATCCTCATTGG-3′	
	Reverse	5′-CCAAACACCAAGCTCATGCGG-3′	
*Il6*	Forward	5′-GCACAACTCTTTTCTCATTTCCACG-3′	Xavier et al. (2013)
	Reverse	5′-GCCTTCCCTACTTCACAAGTCCG-3′	
*Tnfα*	Forward	5′-TTGGGTCTTGTTCACTCCACGG-3′	Winter et al. (2013)
	Reverse	5′-CCTCTTTCAGGTCACTTTGGTAGG-3′	
*Mip2*	Forward	5′-AGTGAACTGCGCTGTCAATGC-3′	Winter et al. (2009)
	Reverse	5′-AGGCAAACTTTTTGACCGCC-3′	

### 2.10 Statistical analysis

The results are expressed as mean ± standard deviation (SD). One-way analysis of variance (ANOVA) was conducted to compare the mean values using IBM SPSS Statistics for Windows, Version 26 (SPSS Inc., USA). Significant differences in EP production and antioxidant activity were determined by Duncan’s multiple range test, with *p*-values less than 0.05 (*p*<0.05) considered statistically significant. In addition, the student’s t-test was used to assess the statistical significance of differences between treatment and control groups regarding the effect of EPs on the cell viability of RAW264.7 cells, invasion assays, and the expression of pro-inflammatory and chemo-attractant cytokine genes.

## 3 Results

### 3.1 Production of fungal EPs

The production of EPs was investigated in MCMB with an initial pH of 6. The results indicated that the seven fungal strains could produce EPs, but at different yields (**Table 2**). Among the strains, the highest yield of EPs was 439.7 ± 37.3 mg/L from *S*. *commune*, which did not show significant differences compared to *G*. *fornicatum* (424.8 ± 35.5 mg/L) and *L*. *sajor*-*caju* (402.7 ± 38 mg/L). On the other hand, *P. sanguineus* exhibited the lowest EP yield at 102.3 ± 16.9 mg/L. The obtained all crude fungal EPs showed water solubilization ability and non-solubilization in ethanol and methanol.

**Table 2:** Fungal species, strain and EP yield.

Fungal species	strains​	Crude EP yields (mg/L)*
*Schizophyllum commune​*	CMU-01​	439.7 ± 37.3 a
*Pycnoporus sanguineus​*	NK0189​	102.3 ± 16.9 c
*Lentinus sajor-caju​*	NK0427​	402.7 ± 38.8 a
*Earliella scabrosa​*	NK0461​	294.9 ± 26.8 b
*Ganoderma fornicatum​*	NK0524​	424.8 ± 35.5 a
*Ganoderma williamsianum​*	NK0540​	260.3 ± 31.8 b
*Favolus tenuiculus​*	NK0550​	239.0 ± 34.6 b

*Results are expressed as mean ± standard deviation. According to Duncan’s multiple range test (*p*<0.05), different letters within the same column are regarded as statistically different.

### 3.2 Optimization of culture conditions for enhanced EP production

#### 3.2.1 Type of liquid culture media

**Figure 1:**
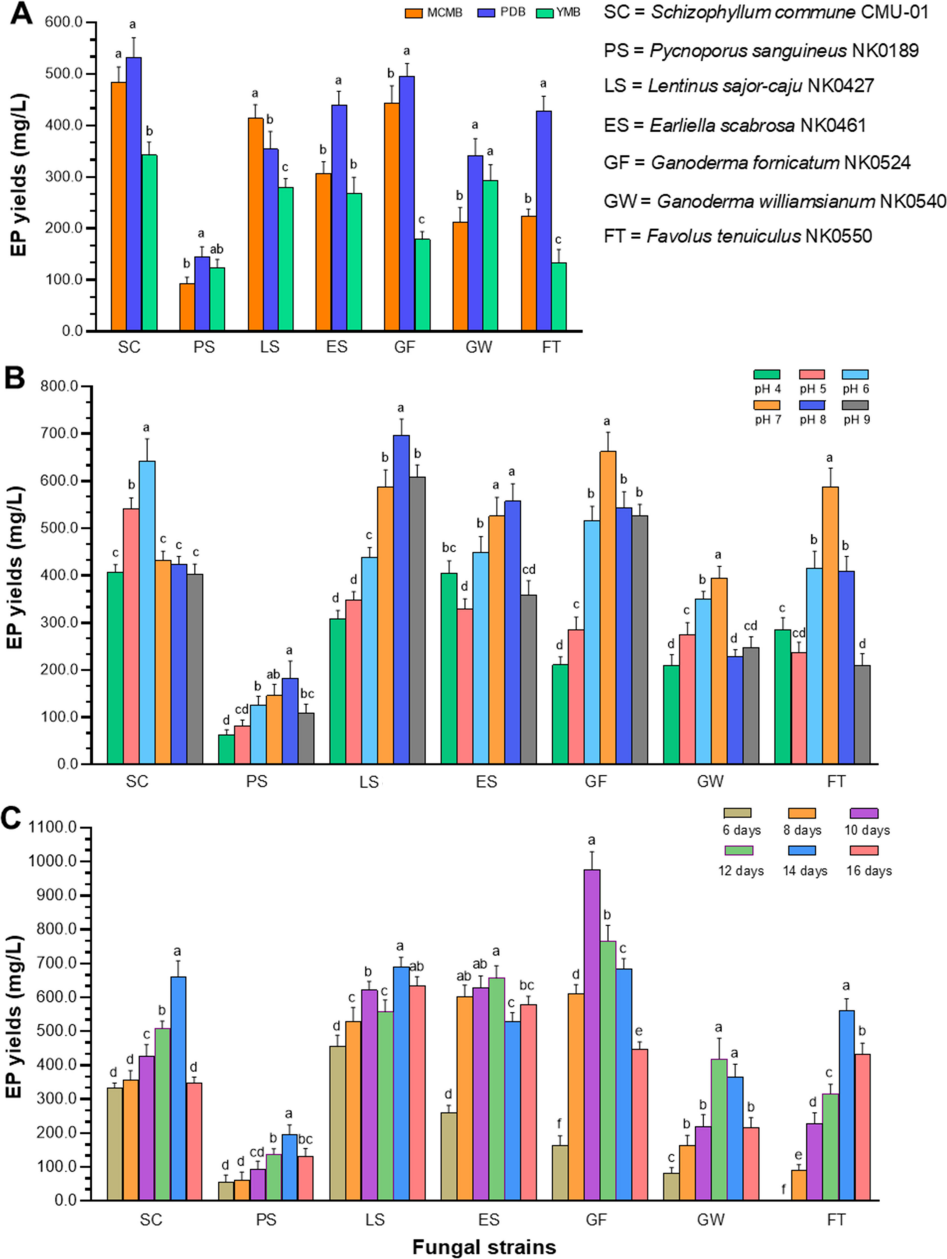
Optimization of culture conditions for enhanced EP production of seven fungal strains. **(A)** EP yields at different liquid culture media. **(B)** EP yields at different initial pH values of the optimal liquid media. **(C)** EP yields at different incubation times. Data are presented as means, and the error bars in each graph indicate the standard deviation (+ SD). Different letters within each graph indicate statistically significant differences by Duncan’s multiple range test (*p*<0.05) in each fungal strain.

Three different types of liquid culture media (MCMB, PDB, and YMB) were used for the production of EPs of the seven fungal strains. The results of the study indicated that six fungi, *S*. *commune*, *G*. *fornicatum*, *E*. *scabrosa*, *F*. *tenuiculus*, *G*. *williamsianum*, and *P*. *sanguineus*, exhibited the highest yield of EPs when cultivated in PDB with respective yields of 533.2 ± 37.5, 496.6 ± 23.9, 439.6 ± 27.0, 427.8 ± 29.3, 341.6 ± 33.0, and 144.0 ± 20.3 mg/L (**Figure 1A**). *Schizophyllum commune* could produce the highest yield of EPs compared to the other fungi. However, only *L*. *sajor*-*caju* demonstrated the highest yield of EPs in MCMB, with a yield of 413.7 ± 26.7 mg/L.

#### 3.2.2 Initial pH values of optimal liquid media

The optimal initial pH values of the culture liquid medium for each fungus, ranging from 4.0 to 9.0, were determined for EP production. The results indicated variations in EP yield among the different pH levels and fungi (**Figure 1B**). *Schizophyllum commune* produced the highest EP yield (643.5 ± 46.1 mg/L) at pH 6 in PDB. Three fungi, including *G. fornicatum, F. tenuiculus* and *G. williamsianum*, produced the highest yield of EPs at pH 7 in PDB. Meanwhile, *E. scabrosa* and *P. sanguineus* demonstrated their highest yields of EPs at pH 8.0 in PDB. It was found that the highest EP yield produced from *L. sajor-caju* was observed at pH 8.0 in MCMB.

#### 3.2.3 Incubation time

The influence of incubation time on EP production was completely examined under controlled conditions, including the suitable culture liquid medium and initial pH value. Notably, *G. fornicatum* exhibited the highest EP yield after 10 days of incubation time, reaching an impressive quantity of 976.5 ± 52.9 mg/L. On the other hand, *E. scabrosa* and *G. williamsianum*, displayed their highest EP yield after 12 days of incubation time, with values of 656.5 ± 36.9 and 419.2 ± 60.9, respectively. Additionally, four fungal strains, namely *L. sajor-caju*, *S. commune*, *F. tenuiculus*, and *P. sanguineus*, demonstrated their highest EP yield after 14 days of incubation (**Figure 1C**). The results of the optimal culture conditions and maximum EP yield for all fungal strains used in this study are presented in **Table 3**.

**Table 3:** The optimal culture conditions of seven fungi in this study for maximum EP production.

Fungal strain​s	Optimal culture conditions			EP production (mg/L)​
	Liquid culture medium​	Initial pH value	Incubation time (days)	
*S. commune* CMU-01	PDB​	6	14	662.0 ± 45.9
*P. sanguineus* NK0189	PDB​	8	14	195.7 ± 28.0
*L. sajor-caju* NK0427	MCMB​	8	14	689.5 ± 28.9
*E. scabrosa* NK0461	PDB​	8	12	656.5 ± 36.9
*G. fornicatum* NK0524	PDB​	7	10	976.5 ± 52.9
*G. williamsianum* NK0540	PDB​	7	12	419.2 ± 60.9
*F. tenuiculus* NK0550	PDB​	7	14	561.6 ± 34.3

MCMB, Mushroom complete medium broth; PDB, Potato dextrose broth.

### 3.3 Structural characterization of crude fungal EPs by FT-IR spectroscopy

**Figure 2:**
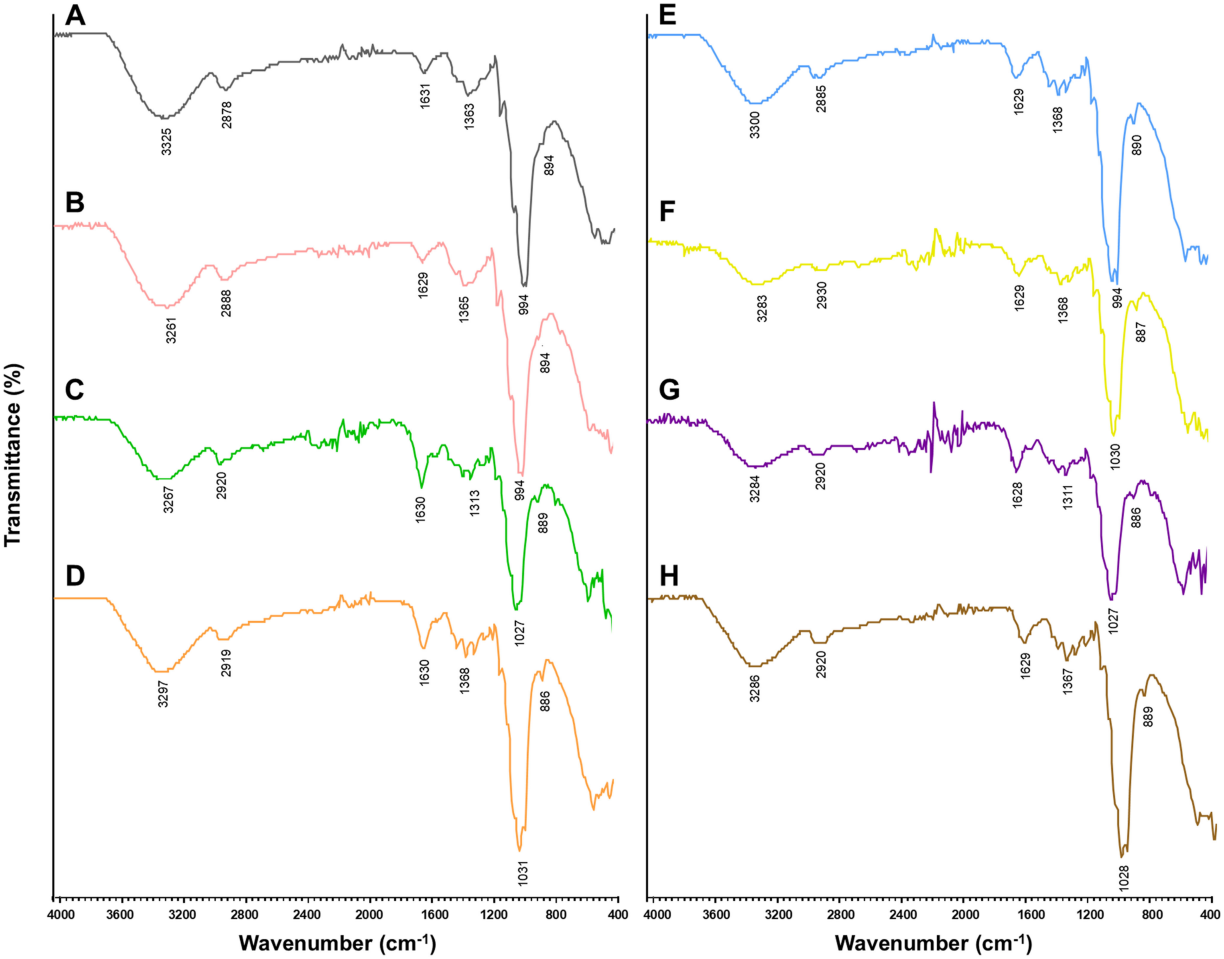
Structural characteristics by FT-IR spectroscopy of standard EPs **(A)** schizophyllan and crude fungal EPs; **(B)** *S. commune* CMU-01, **(C)** *P. sanguineus* NK0189, **(D)** *L. sajor-caju* NK0427, **(E)** *E. scabrosa* NK0461, **(F)** *G. fornicatum* NK0524, **(G)** *G. williamsianum* NK0540, and **(H)** *F. tenuiculus* NK0550.

FT-IR analysis was conducted to identify the chemical functional groups present in the crude EPs of the seven fungi. The FT-IR spectra of the crude fungal EPs and the schizophyllan standard are shown in **Figure 2** and **Table 4**. The results indicated that the FT-IR spectra of all crude fungal EPs exhibited typical carbohydrate patterns similar to those of schizophyllan. The broad peak in the range of 3,252.1 to 3,300.1 cm^-1^ was attributed to the hydroxyl (-OH) stretching vibration of the EPs and the main functional group of polysaccharides (Radzki et al., 2016). The peak in the region of 2,885.0 to 2,930.0 cm^-1^ corresponded to the stretching vibration of C-H of the aldehyde and methylene group (CH_2_) from saturated carbon (Tabibzadeh et al., 2022). An absorption peak recorded in the range of 1,628.0 to 1,631.8 cm^-1^ was assigned to carbonyl (C=O) stretching and C=O, C=N stretching vibration of amide (RCONH_2_) (Lima et al., 2016; Sountharavallee et al., 2023). The bands between 1,313.8 and 1,368.5 cm^-1^ indicated bending vibration of C-H and C-C stretching of aliphatic chains (Zhang et al., 2017). Additionally, the strong absorbance in the range of 994.2 to 1,033.2 cm^-1^ in the spectra of all crude fungal EPs represented the characteristic absorption peak of polysaccharides. These strong absorption bands were attributed to the stretching vibration of C-O, C-O-C, and C-O-H linkages corresponding to the *α*-glycosidic linkages (Ren et al., 2015; Li et al., 2017). Furthermore, the distinct characteristic absorption at approximately 885.9 to 895.9 cm^-1^ indicated the *β*-glycosidic bonds from sugar components (Du et al., 2017; Jaroszuk-Ściseł et al., 2020).

**Table 4:** Structural characteristics of the crude fungal EPs by FT-IR spectroscopy.

Wavenumber (cm^-1^)	Functional group/Characteristics​
3,252.1 to 3,300.1	Hydroxyl group (-OH) of the polysaccharides​ related to inter-residue hydrogen linkages
2,885.0 to 2,930.0	C-H bonds of aldehyde groups and methylene groups (CH_2_) from saturated carbon
1,628.0 to 1,631.8	Carbonyl group (C=O) and C=O, C=N stretching vibration of amide (RCONH_2_)
1,313.8 to 1,368.5	​Bending vibration of C-H and stretching vibration of C-C from aliphatic chain
994.2 to 1,033.2	Oxygen groups of C-O, C-O-C, C-O-H and *α*-glycosidic linkages ​of polysaccharides
885.9 to 895.9	*β*-glycosidic linkages​ of polysaccharides

### 3.4 Monosaccharide compositions

The monosaccharide compositions of the crude fungal EPs were identified through the HPLC. The relation time of allose, allulose, fructose, mannose, glucose, rhamnose, and xylose were 9.8, 11.4, 12.4, 13.6, 14.7, 17.2, and 33.2 min. Our findings revealed that these seven fungal EPs are heteropolysaccharides. The monosaccharide compositions in each fungal EPs differed between fungal species (**Table 5**). The result indicated that glucose was found to be the main monosaccharide composition of all fungal EPs. In addition, fructose was the minor composition of the fungal EPs obtained from *P. sanguineus*, *E*. *scabrosa*, and *F*. *tenuiculus*. Whereas, allose was found as a minor component in the EPs obtained from *L. sajor-caju* and *G. williamsianum*. Allulose was found as a minor component in the EPs obtained from *G. fornicatum*. Xylose was only found in EPs obtained from *L*. *sajor*-*caju*. Rhamnose was found in EPs obtained from *F*. *tenuiculus* and *G*. *fornicatum*. EPs obtained from *G. fornicatum* and *G*. *williamsianum* were comprised of allulose. However, mannose was not found in all fungal EPs.

**Table 5:** Monosaccharide compositions of the seven fungal EPs.

Fungal strains	Amount of monosaccharide compositions (mg/g)					
	Rhamnose	Allulose	Xylose	Fructose	Allose	Glucose
*S. commune* CMU-01	–	–	–	–	7.28	831.43
*P*. *sanguineus* NK0189	–	–	–	51.47	–	84.68
*L*. *sajor*-*caju* NK0427	–	–	58.36	42.62	74.97	77.14
*E*. *scabrosa* NK0461	–	–	–	111.92	–	117.68
*G*. *fornicatum* NK0524	29.30	33.85	–	–	–	143.15
*G*. *williamsianum* NK0540	–	105.16	–	–	115.21	129.78
*F*. *tenuiculus* NK0550	146.97	–	–	193.67	–	219.05

### 3.5 Antioxidant activities of crude EPs

The antioxidant activities of crude EPs obtained from seven fungal strains were evaluated using two assays, including ABTS and DPPH radical scavenging activities. The result demonstrated that EPs from all fungi have positive antioxidant potential activity. In this study, the IC_50_ value was used to express the radical scavenging abilities of DPPH and ABTS as presented in **Table 6**. The lower IC_50_ value indicated the higher antioxidant activity. In terms of the ABTS assay, the crude EPs of *E. scabrosa* exhibited the highest ABTS free radical scavenging activity, with the IC_50_ value of 4.450 ± 0.014 mg/mL. The crude EPs from *P. sanguineus*, *G. fornicatum*, *S. commune*, *F. tenuiculus*, and *L. sajor-caju* exhibited IC_50_ values of 7.562 ± 0.078, 8.956 ± 0.358, 9.543 ± 0.363, 9.950 ± 0.212, and 10.218 ± 0.298 mg/mL, respectively. On the other hand, the crude EPs obtained from *G. williamsianum* displayed the lowest ABTS free radical scavenging activity, with an IC_50_ value of 13.292 ± 0.519 mg/mL.

**Table 6:** The antioxidant potential of crude EPs from seven fungal strains.

Crude fungal EPs	IC_50_ value (mg/mL)*	
	ABTS radical scavenging	DPPH radical scavenging
*S. commune* CMU-01	9.543 ± 0.363 e	1.420 ± 0.012 b
*P. sanguineus* NK0189	7.562 ± 0.078 c	1.525 ± 0.028 c
*L. sajor-caju* NK0427	10.218 ± 0.298 f	1.699 ± 0.010 de
*E. scabrosa* NK0461	4.450 ± 0.014 b	1.659 ± 0.018 d
*G. fornicatum* NK0524	8.956 ± 0.358 d	1.674 ± 0.065 de
*G. williamsianum* NK0540	13.292 ± 0.519 g	1.713 ± 0.007 e
*F. tenuiculus* NK0550	9.950 ± 0.212 ef	1.510 ± 0.015 c
Gallic acid (positive control)	0.043 ± 0.010 a	0.005 ± 0.001 a

*Results are expressed as mean ± standard deviation. The distinct letters within the same column are considered significantly distinct based on Duncan’s multiple range test (*p* < 0.05).

Regarding the DPPH assay, the crude EPs produced from *S. commune* exhibited the highest DPPH free radical scavenging activity, with an IC_50_ value of 1.420 ± 0.012 mg/mL. In addition, the crude EPs from *F. tenuiculus*, *P. sanguineus*, *E. scabrosa*, *G. fornicatum*, and *L. sajor-caju* exhibited IC_50_ values of 1.510 ± 0.015, 1.525 ± 0.028, 1.659 ± 0.018, 1.674 ± 0.065, and 1.699 ± 0.010 mg/mL, respectively. Conversely, the crude EPs obtained from *G. williamsianum* displayed the lowest DPPH free radical scavenging activity, with an IC_50_ value of 1.713 ± 0.007 mg/mL. However, it was found that gallic acid exhibited greater ABTS and DPPH radical scavenging activities than the crude fungal EPs, with exceptional IC_50_ values of 0.043 ± 0.010 and 0.005 ± 0.001 mg/mL, respectively.

### 3.6 Effect of crude fungal EPs on the cell viability of RAW264.7 cells

**Figure 3:**
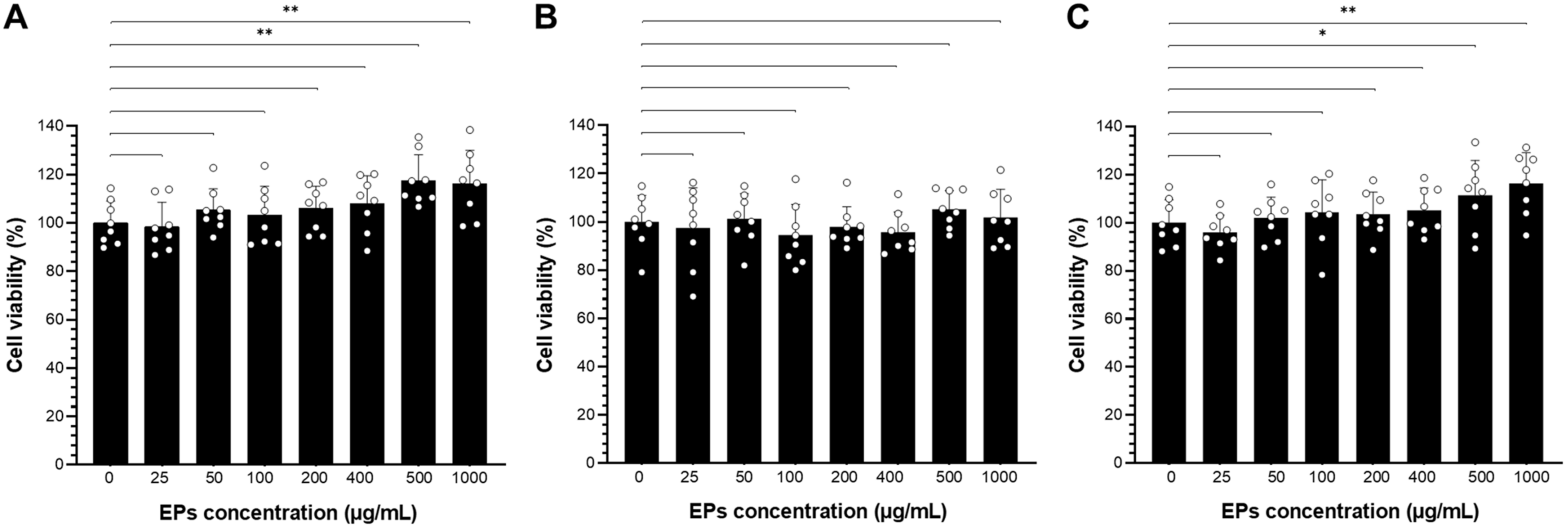
The cell viability of fungal EPs-treated RAW264.7 cells was determined by 3-(4,5-dimethylthiazol-2-yl)-2,5-diphenyltetrazolium bromide (MTT) assay. Murine macrophage cell lines were treated with the fungal EPs of *S*. *commune* CMU-01; SC-EPs **(A)**, *G. fornicatum* NK0524; GF-EPs **(B)**, and *L*. *sajor*-*caju* NK0427; LS-EPs **(C)** at different final concentrations (25, 50, 100, 200, 400, 500, and 1,000 μg/mL) for 24 hours exposure. Data are presented as means, and the error bars in each graph indicate the standard deviation (+ SD). One scatter dot represents each replicate. The * and ** represent significant differences with *p*-values < 0.05 and 0.01, respectively, compared to the EPs-untreated control group (0 μg/mL), as determined by Student’s *t*-test.

Next, we investigated the role of fungal EPs on the macrophage, the important innate immune cell in mammals; the cytotoxicity of fungal EPs on murine macrophage (RAW264.7) was performed by the MTT assay. The three selected crude fungal EPs from *S*. *commune* (SC-EPs), *G*. *fornicatum* (GF-EPs), and *L*. *sajor*-*caju* (LS-EPs) at various concentrations (25–1000 µg/mL) were incubated for 24 h. Three selected fungal EPs revealed non-cytotoxicity to RAW264.7 cells at concentrations in the range of 25-1,000 μg/mL as compared with the EPs-untreated control group (**Figure 3**). Interestingly, RAW264.7 cells treated with SC-EPs at concentrations of 500 and 1,000 µg/mL showed a significant enhancement of cell viability (*p* < 0.01), as shown in **Figure 3A**. Similarly, LS-EPs-treated RAW264.7 cells showed increasing effects on cell viability with concentrations of the polysaccharides at 500 and 1,000 µg/mL, in a dose-dependent manner, with *p*-values of < 0.05 and < 0.01, respectively (**Figure 3C**). However, there were no significant differences in the RAW264.7 cells treated with GF-EPs compared to the EPs-untreated control group (**Figure 3B**).

### 3.7 Pretreatment with fungal EPs increased macrophage-killing activity against *Salmonella enterica* serovar Typhimurium infection in a dose-dependent manner

#### 3.7.1 Invasion assay

**Figure 4:**
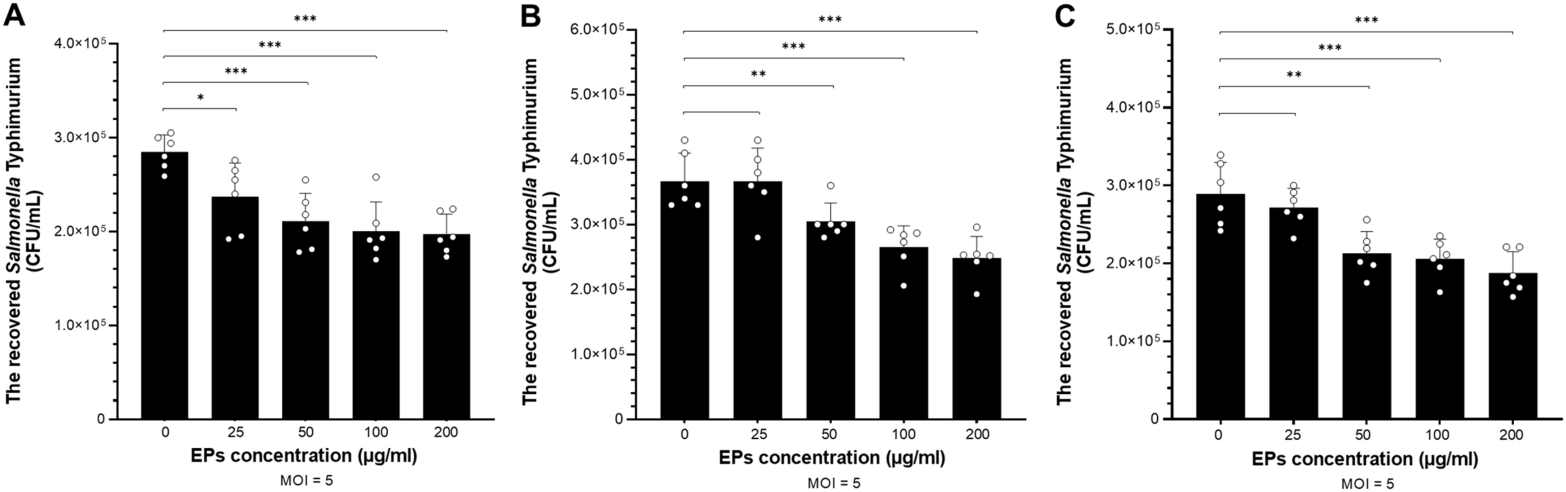
The viable number of STM recovered from macrophages was enumerated to assess the immunomodulatory effect of fungal EPs on the macrophage against STM. The invasion (gentamicin protection) assay was conducted in RAW264.7 murine macrophage cell lines. The cells were pre-treated with the fungal EPs of *S*. *commune* CMU-01; SC-EPs **(A)**, *G. fornicatum* NK0524; GF-EPs **(B)**, and *L*. *sajor*-*caju* NK0427; LS-EPs **(C)** at different final concentrations of 25, 50, 100, and 200 μg/mL. After 24 hours of fungal EP treatment, the cells were exposed to STM with an MOI of 5 for 1 hour. Data are presented as means, and the error bars in each graph indicate the standard deviation (+ SD). The *, **, and *** represent significant differences with *p*-values < 0.05, 0.01, and 0.001, respectively, compared to the EPs-untreated control group (0 μg/mL), as determined by Student’s *t*-test.

We investigated the effect of fungal EPs on the immunomodulatory effect of fungal EPs on macrophage against *Salmonella* infection. Macrophages were pre-treated with three selected fungal EPs (*S*. *commune* CMU-01, *G*. *fornicatum* NK0524, and *L*. *sajor*-*caju* NK0427) at concentrations of 25, 50, 100, and 200 µg/mL for 24 hours prior to infection with *Salmonella enterica* serovar Typhimurium. After pretreatment with fungal EPs, RAW264.7 cells were infected with STM (MOI 5) for 1 h. After the elimination of extracellular bacteria by gentamicin sulfate treatment, the macrophage cells were collected and lysed. Then, the viable number of recovered STM from the intracellular macrophages was enumerated on LB agar supplemented with nalidixic acid. The results indicated that the three fungal EPs promoted the elimination of foreign bacteria entering macrophage cells and attenuated the survival of *Salmonella* in intracellular macrophage by reducing the viable number of recovered STM in a dose-dependent manner (**Figure 4**).

Compared with the EPs-untreated group, SC-EPs-treated RAW264.7 cells were the only group in which the number of recovered STM from intracellular macrophage had significantly decreased (*p*<0.05) at a low concentration (25 µg/mL), as shown in **Figure 4A**. At higher concentrations for treatment of SC-EPs (50–200 µg/mL), RAW264.7 cells exhibited a significant reduction in the number of recovered STM (*p*<0.001). Additionally, RAW264.7 macrophages treated with GF-EPs and LS-EPs also showed a significantly decreased viable number of survived STM in intracellular macrophages with a dose-dependent relationship. In other words, GF-EPs and LS-EPs-treated RAW264.7 cells at the concentration of 50 µg/mL significantly reduced the number of recovered STM (*p*<0.01). Meanwhile, pretreatment of GF-EPs and LS-EPs (100 and 200 µg/mL) inhibited the macrophage intracellular survival of STM and decreased the number of recovered STM significantly (*p*<0.001), as shown in **Figures 4B**, **C**. These results indicated that fungal EPs enhance the killing activity of RAW264.7 in a concentration-dependent manner. The fungal EPs from all mushroom strains at concentrations of 50 µg/mL significantly reduced the number of invaded STM.

#### 3.7.2 Pro-inflammatory and chemo-attractant cytokine genes expression of EPs-treated and STM-infected RAW264.7 cells

**Figure 5:**
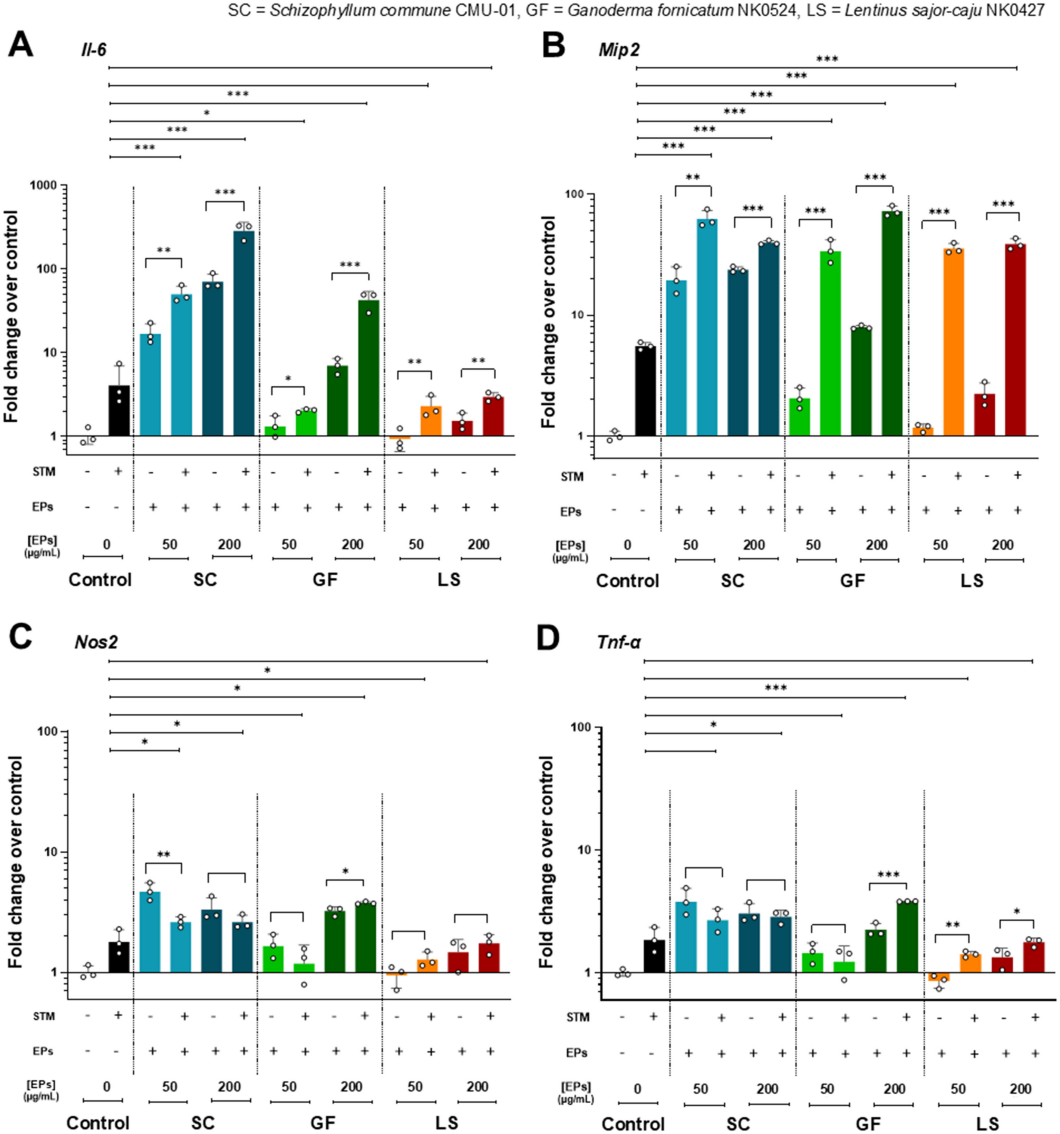
The pro-inflammatory cytokine gene expressions in the EPs-pretreated and STM-infected RAW264.7 cells. Fold change of gene expression of RAW264.7 pretreated with fungal EPs of *S*. *commune* CMU-01 (SC-EPs), *G. fornicatum* NK0524 (GF-EPs), and *L*. *sajor*-*caju* NK0427 (LS-EPs) at 24 hours and infected by STM for 1 hour compared to the untreated cells. The mRNA expression of **(A)** *Il6*, **(B)** *Mip2*, **(C)** *Nos2*, and **(D)** *Tnfα*. All genes were normalized with the PBS control (EPs and STM-untreated RAW264.7 cells) and analyzed with a comparative threshold cycle (C_T_) method over the housekeeping gene (*Gapdh*). Data are presented as geometric means, and the error bars in each graph indicate the geometric standard deviation (+ SD). The *, **, and *** represent significant differences with *p*-values < 0.05, 0.01, and 0.001, respectively, compared with the control (EPs-untreated and STM-infected RAW264.7 cells) and during the same group, as determined by Student’s t-test.

The immunomodulatory effect of fungal EP treatment on RAW264.7 murine macrophages was determined by the expression of pro-inflammatory and chemo-attractant cytokine genes. After pretreatment with three selected fungal EPs at concentrations of 50 and 200 µg/mL for 24 hours, RAW264.7 cells were treated and infected by STM with an MOI of 5 for 1 hour. Then, the cells were collected and extracted for total RNA. The mRNA of pro-inflammatory cytokine genes was amplified and evaluated for expression compared with EPs-untreated and STM-treated control groups in the four genes of interest (*Il6*, *Mip2*, *Nos2*, and *Tnfα*) by RT-qPCR. As depicted in **Figures 5A, B**, RAW264.7 macrophages pre-treated with three fungal EPs and then infected with STM exhibited enhanced expression of cytokines IL-6, MIP-2, iNOS, and TNF-*α* compared to those from the EPs-untreated and STM-infected control group. These results suggest that the presence of fungal EPs can enhance the production of pro-inflammatory cytokines. However, this enhancement depends on the source of fungal EPs and the final concentration of fungal EPs in cell culture.

RAW264.7 cells pre-treated with SC-EPs and infected with STM showed significantly enhanced (*p*<0.001) expression of two pro-inflammatory cytokine genes (*Il6* and *Mip2*) in a dose-dependent manner compared to the STM-infected and EPs-untreated control group (**Figures 5A, B**). Similarly, they exhibited a significant increase (*p*<0.05) in the expression of two pro-inflammatory cytokine genes (*Nos2* and *Tnfα*), as shown in **Figures 5C, D**. The expression of *Il6*, *Mip2*, and *Tnfα* in GF-EPs-pretreated RAW264.7 cells at a concentration of 200 µg/mL with STM infection significantly increased (*p*<0.001) compared to the STM-infected and EPs-untreated control group, as shown in **Figures 5A, B, D**. Additionally, RAW264.7 cells pre-treated with 50 µg/mL of GF-EPs and infected with STM exhibited significantly enhanced expression of *Il6* (*p*<0.05) and *Mip2* (*p*<0.001), as shown in **Figure 5A**. Likewise, pretreatment of RAW264.7 macrophages with GF-EPs at concentrations of 50 and 200 µg/mL resulted in mRNA expression of *Il6*, *Mip2*, *Nos2*, and *Tnfα* in a concentration-dependent manner.

Pretreatment of RAW264.7 cells with 50 and 200 µg/mL of LS-EPs, followed by STM infection, significantly increased (*p*<0.001) the expression of *Mip2* compared to the STM-infected and EPs-untreated control group, as shown in **Figure 5B**. The expression of *Il6*, *Nos2*, and *Tnfα* in LS-EPs-pretreated RAW264.7 cells with STM infection was lower than in the STM-infected control group but higher than in the EPs-untreated control group, as shown in **Figures 5A, C, D**. However, the expression of pro-inflammatory cytokine and chemo-attractant cytokine genes in LS-EPs-treated RAW264.7 cells increased in a dose-dependent relationship. Overall, pretreatment of RAW264.7 cells with fungal EPs at concentrations of 50 or 200 µg/mL without STM infection induced higher expression of the cytokine genes compared to EPs-untreated RAW264.7 cells. Notably, RAW264.7 cells pretreated with EPs and infected with STM exhibited a higher expression of pro-inflammatory cytokine genes than RAW264.7 macrophages treated only with EPs. These data exhibited the immunomodulatory effects of fungal EPs obtained from three fungi (*S*. *commune*, *G*. *fornicatum*, and *L*. *sajor*-*caju*). Pretreatment of macrophage with fungal EPs significantly stimulated the expression of pro-inflammatory genes when exposed to STM infection and reduced intracellular STM population.

## 4 Discussion

Nowadays, research aimed at enhancing the production of fungal EPs from each fungal species and strain focuses on optimizing conditions to achieve the highest yield. In this present study, seven fungal strains (*S. commune* CMU-01, *P. sanguineus* NK0189, *L. sajor-caju* NK0427, *E. scabrosa* NK0461, *G. fornicatum* NK0524, *G. williamsianum* NK0540, and *F. tenuiculus* NK0550) were found to produce EPs in MCMB with a pH of 6. The highest EP yield was produced by *S. commune*. This study is consistent with previous research, which indicates that fungi in the genera *Ganoderma*, *Lentinus*, *Pycnoporus*, and *Schizophyllum* have the ability to produce EPs in liquid cultivation (Jeong et al., 2009; Hao et al., 2010; Cao et al., 2014; Osińska-Jaroszuk et al., 2015). Interestingly, this study is the first to report EP production in the genera *Earliella* and *Favolus*. Our results found that the optimal conditions for EP production in each fungal strain varied based on the type of liquid culture medium. Among the three different liquid media (MCMB, PDB, and YMB), the maximum EP yield produced by *S. commune*, *G. fornicatum*, *G. williamsianum*, *E. scabrosa*, *F. tenuiculus*, and *P. sanguineus* was obtained in PDB, while the maximum EP yield of *L. sajor-caju* was found in MCMB. This result is supported by the findings of several previous studies, which reported that the production of fungal EPs from each strain in liquid cultivation is influenced by the type of cultivation medium (Elisashvili et al., 2009, 2012; Mahapatra and Banerjee, 2013). For example, Kim et al. (2002) reported that MCMB served as the optimal culture medium for the highest EP production in *Agrocybe cylindracea*, *Collybia maculata*, and *G*. *lucidum*, while PDB was the optimal medium for the highest EP production in *Trametes versicolor*, *Phellinus linteus*, *Phellinus pini*, and *Pleurotus ostreatus*. A study by Kizitska et al. (2024) highlighted that PDB was suitable for maximizing EP yield of *Fomitopsis betulina*. In contrast, Tavares et al. (2005) and Ahmed et al. (2011) found that YMB was found to be the most effective for EP production from *Trametes versicolor*. Therefore, the optimization of the medium type should be taken into consideration in order to achieve the highest yield of fungal EPs.

The pH value of the liquid medium and the incubation period had a significant effect on EP production in fungi. In this study, it was observed that the pH of the liquid medium has a significant effect on the production of EPs. The highest EP yield for each fungal strain varied among the different pH levels. *Schizophyllum commune* produced the highest EP yield at pH 6 in PDB. Three fungi, including *G. fornicatum*, *F. tenuiculus* and *G. williamsianum*, produced the highest yield of EPs at pH 7. Meanwhile, *E. scabrosa*, *L. sajor-caju* and *P. sanguineus* demonstrated their highest yields of EPs at pH 8.0. These results were in accordance with the findings of previous studies which had reported that the optimal pH value of the liquid medium range from 4.0–9.0 depending on both the fungal species and on the fungal strain (Chen et al., 2008; Osman et al., 2009; Mahapatra and Banerjee, 2013; Osińska-Jaroszuk et al., 2015). For example, the optimal pH for achieving the highest EP production from *Lignosus rhinoceros* and *G. resinaceum* was pH 6 (Lai et al., 2014; Kim et al., 2006). Hamedi et al. (2007) found that *Agaricus blazei* produced the highest EPs at a pH of 7. In addition, Thai and Keawsompong (2019) reported that a pH value of 6.5 supported the maximum EP production yield in *Tricholoma crassum*. *Cordyceps militaris and Tremella fuciformis* produced the highest EPs at a pH of 8 (Kim and Yun, 2005; Cho et al., 2006). In this study, the incubation period for the fungal EP production was investigated. After 10 days of incubation, *G. fornicatum* produced the highest yield of EPs. However, after 12 days of incubation, *G. williamsianum* and *E. scabrosa* exhibited their highest EP production. The largest yield of EPs was observed by *F. tenuiculus*, *L. sajor-caju*, *P. sanguineus*, and *S. commune* after 14 days of incubation. The results of this study are consistent with previous research, which found that the optimal incubation period for achieving the highest fungal EP production varied depending on the fungal species and strains (Nehad and El-Shamy, 2010; Mahapatra and Banerjee, 2013; Jia et al., 2015; Osińska-Jaroszuk et al., 2015). Rosado et al. (2003) and Long et al. (2021) reported that *Pleurotus ostreatoroseus* and *G*. *cantharelloideum* yielded the highest amounts of EP production after 7 days of incubation. A study of Jeong et al. (2009) found that the maximum yield of EPs from *G. applanatum* was achieved after 12 days. Sasidhara and Bakki (2014) investigated the production of EPs in *G. lucidum* and discovered that the maximum yield of EPs was produced after 14 days of incubation. Therefore, our findings highlight the optimal conditions required for each fungal strain to achieve its maximum EP yield. Knowing the optimal cultivation period for fungal EP production helps maximize yield, improve cost efficiency, and develop effective strategies for industrial production. However, several other factors, including aeration, temperature, carbon sources, and nitrogen sources, significantly influence fungal EP production. These parameters can affect not only the yield but also the composition and bioactivity of the produced EP. Future research should focus on optimizing these factors for each specific fungal strain to enhance EP production and biological efficiency.

The functional groups of the seven fungal EPs in this study were identified through FT-IR spectrum analysis. Their spectra unveiled functional groups, confirming the typical patterns of polysaccharides. This included broad peaks indicating hydroxyl (-OH) groups, C-H bonds from aldehyde groups, methylene (CH_2_) groups, carbonyl (C=O) groups, and C-H and C-C bonds from aliphatic hydrocarbons. Strong peaks were observed for polysaccharide features, including C-O, C-O-C, and C-O-H linkages. A comparison of the obtained peaks indicated the presence of both *α* and *β* glycosidic bonds, further confirming the classification of these fungal EPs as carbohydrates. Moreover, the presence of carbonyl groups in these EPs suggests their potential inclusion as part of amide (RCONH_2_) groups, indicating that the fungal crude EPs are protein-complex polysaccharides. These characteristic properties of the IR spectrum of these fungal EPs were similar to those of previous reports on the properties of fungal EPs (Zheng et al., 2014; Hu et al., 2017; Usuldin et al., 2020; Chen et al., 2023a; Fornal et al., 2023). For monosaccharide composition analysis, the obtained fungal EPs were heteropolysaccharides, with glucose as the major monosaccharide and minor amounts of fructose, allose, rhamnose, allulose, and others. Similarly, previous studies have reported that glucose is the most common monosaccharide in several fungal EPs, along with other monosaccharides, including arabinose, galactose, fucose, mannose, rhamnose, and xylose (Peng et al., 2015; Cao et al., 2019; Mehmood et al., 2019; Xu et al., 2019a; Long et al., 2021). Moreover, several previous studies reported that the monosaccharide composition of EPs can be significantly influenced by nutrient (carbon and nitrogen sources) availability and liquid culture conditions, especially the carbon source (Chen et al., 2015; Wu and Liang, 2018; Wu et al., 2021).

In this study, the EPs obtained from the seven fungal strains demonstrated positive antioxidant activity in both the DPPH and ABTS radical scavenging assays, which are commonly used to assess antioxidant activity *in vitro*. Several studies have documented the antioxidant activities of fungal EPs, quantifying these properties through IC_50_ values employing DPPH and ABTS radical scavenging activity assays. The IC_50_ value represents the concentration required to scavenge 50% of free radicals. Our study demonstrated that the fungal EPs exhibited antioxidant activity with IC_50_ values ranging from 4.45 to 13.29 mg/mL for ABTS assay and 1.42 to 1.71 mg/mL for DPPH assay. These findings are consistent with previous studies reporting that EPs from basidiomycetous fungal species in genera such as *Agrocybe*, *Ganoderma*, *Hygrophoropsis*, *Inonotus*, *Laetiporus*, *Nothophellinus*, *Pleurotus*, *Pseudoinonotus*, *Rigidoporus*, *Trametes*, and *Tremella* exhibit antioxidant activities, playing a significant role as free radical scavengers, with the activity of EPs varying according to the fungal species and strains (Lung & Huang, 2012; Zhang et al., 2015; Zhao et al., 2017; Jia et al., 2018; Xu et al., 2019b; Bai et al., 2020; Huang et al., 2020; Zheng et al., 2020; Debnath et al., 2021; Zhang et al., 2023; Gallo et al., 2024). For instance, EPs obtained from *C. sinensis* showed IC_50_ values for ABTS activity ranging from 1.027 to 2.953 mg/mL (Ho-Thi et al., 2016; Nguyen et al., 2021). In contrast, the IC_50_ values for ABTS activity of EPs obtained from *C. cicadae* and *C. gracilis* were 6.38 and 7.08 mg/mL, respectively (Sharma et al., 2015a, b). The EPs of *C. cicadae*, *Hericium coralloides*, *P. flabellatus*, and *Tricholoma crissum* had IC_50_ values for DPPH activity of 7.32, 6.59, 0.93 to 2.25, and 0.338 mg/mL, respectively (Sharma et al., 2015a; Thai and Keawsompong, 2019; Nguyen et al., 2021; Tabibzadeh et al., 2022). Prior to this present study, the antioxidant activities of EPs obtained from several basidiomycetous fungi (e.g. *Fomitopsis meliae*, *G. lucidum*, *Inonotus obliquus* and *Stropharia rugosoannulata*) have been reported from a variety of assays employing different mechanisms including lipid peroxidation, metal chelation, reducing power and scavenging activity, among others (Xu et al., 2014; Liu and Zhang, 2018; Hao et al., 2023; Prajapati et al., 2023). However, variations in the assays themselves, and the results they express, make it difficult to compare the outcomes obtained in this study with those of previous studies. Several previous studies have reported that EPs exhibit potent antioxidant activities, which are associated with their structural and chemical characteristics, including the presence of hydroxyl groups (-OH) and other specific functional groups in the structure of EPs, such as -COOH, C=O, and -O-. These groups can donate hydrogen ions or electrons to reduce free radicals, leading to the formation of more stable compounds, react with free radicals to terminate radical chain reactions, and stabilize free radicals through direct binding (Leung et al., 2009; He et al., 2012; Mao et al., 2014; Sun et al., 2015; Chen et al., 2017). Further studies are needed to elucidate the detailed chemical structure and molecular characteristics of the EPS investigated in this study. Advanced analytical techniques such as nuclear magnetic resonance (NMR) spectroscopy and size-exclusion chromatography should be employed to determine glycosidic linkages, molecular weight, and branching patterns.

Fungal EPs are present as alternative substances that have no toxicity for the activation of macrophages. These EPs obtained from different species of fungi exhibit properties that can modulate the immune response, thereby stimulating the activation of macrophages without causing any harmful effects on their function or viability. In this study, the effect of fungal EPs on the cell viability of RAW264.7 cells was examined by exposing the cells to three selected fungal EPs at different concentrations. The MTT assay indicated that the fungal EPs displayed no cytotoxic effects on the cells when present at concentrations ranging from 25 to 1,000 μg/mL, with some outstanding promotion of cell proliferation. In consistent with Zhang et al. (2022), the polysaccharides of *Morchella sextelata* showed no cytotoxicity at concentrations of 12.5 to 800 μg/mL and had a positive effect on the cell viability of RAW264.7 cells by significantly promoting cell proliferation. Similarly, crude and fractions of *S*. *commune* polysaccharides also significantly improved the proliferation activity of RAW264.7 cells, indicating that these certain concentrations of fungal polysaccharides were nontoxic to macrophages (Yelithao et al., 2019). Many studies have investigated the effect of fungal EP treatment on the RAW264.7 cells. It was found that fungal EPs not only promote cell viability, but they also differentiate the macrophage cells. Consistent with a study by Yang et al. (2023), fungal EPs derived from *Cordyceps cicadae* were found to stimulate growth, increase the density, and alter the morphology of the RAW264.7. The cells were larger and formed clusters at a higher concentration of EPs compared to the EPs-untreated control group. In addition, crude polysaccharides from *Calocybe indica* induced morphological changes in RAW264.7 macrophage cells, affecting treated cells to exhibit dendritic extensions in a dose-dependent manner (Ghosh et al., 2021). Fungal polysaccharides extracted from *Dictyophora rubrovolvata* have been found to promote the differentiation of RAW264.7 cells (Bao et al., 2023). Moreover, polysaccharides derived from *Pleurotus tuber*-*regium* were found to activate enzymes accountable for the maturation and proliferation of mice peritoneal macrophages. The treatment of fungal polysaccharides resulted in an augmentation of primary and secondary lysosomes in the cytoplasm, along with an increased abundance of ribosomes and dense chromatin in the nucleus of polysaccharide-treated cells (Wu et al., 2014). Interestingly, *Inonotus obliquus* polysaccharides have been found to promote cell proliferation in mouse splenocytes. This indicates that the fungal polysaccharides activate not only RAW264.7 macrophage cells but also other immune cells (Won et al., 2011). For a more thorough understanding of the cellular responses and possible underlying mechanisms of EPs in this study, future research should include further investigations into morphological alterations and stress markers, such as reactive oxygen species (ROS) production and apoptosis-related proteins, in macrophages.

Fungal polysaccharides are complex biomacromolecules and have been the focus of scientific investigations for their remarkable capacity to augment the process of phagocytosis in macrophage. Macrophages are the important innate immune cells in mammals. Upon encountering pathogenic triggers or suffering injury, the process of phagocytosis serves as the early stage in the immune reaction of macrophages toward several pathogens, especially bacteria (Weiss and Schaible, 2015). Phagocytosis is a crucial function of macrophages wherein they engulf and digest the pathogens. The effectiveness of macrophages in engulfing particles was assessed based on their uptake of neutral red or fluorescein isothiocyanate (FITC)-dextran after prior treatment with fungal polysaccharides (Zhang et al., 2008; Wan et al., 2023). Several studies have demonstrated that fungal EPs can boost the phagocytic function of RAW264.7 *in vitro*. Pretreatment with fungal EPs isolated from *Cordyceps militaris* and *Paecilomyces cicadae* TJJ1213 has been shown to increase the ingestion step in macrophages (Lee et al., 2015; Tian et al., 2022), as well as fungal EPs from a liquid culture of *L*. *edodes* were reported to be implicated in the enhancement of phagocytosis (Lee et al., 2008). Markedly, the phagocytosis activities of macrophages induced by fungal polysaccharides showed a concentration-dependent manner in the range of 50 to 200 µg/mL (Zhang et al., 2022).

In our study, we examined the impact of fungal EPs on the intracellular survival of *Salmonella* within macrophage cells. The RAW264.7 cells were pre-treated with three selected fungal EPs at various concentrations before being infected with STM. *Salmonella* is a diverse species of Gram-negative bacteria that can cause acute gastroenteritis in humans and animals. *Salmonella* infections have a significant impact on public health and the food industry globally due to their potential virulence factors and the increase in mutlidrug-resistant (MDR) strains. The bacteria have evolved mechanisms to subvert the host’s immune system and colonize the host, employing the specialized structure named “Type III secretion systems (T3SS)” to inject effector proteins into the gut epithelium and macrophage cytosol. These proteins manipulate host cell functions, aiding the establishment of intracellular niches and enhancing bacterial survival (Buckner et al., 2011). *Salmonella* resides within modified phagosomes known as *Salmonella*-containing vacuoles (SCVs), preventing bacterial degradation and enabling survival within phagosomal compartments (Kombade and Kaur, 2021). Additionally, *Salmonella*-induced filaments (SIFs) are membranous structures formed by the bacterium, contributing to a sheltered intracellular environment for replication within macrophages and evading host immune responses by altering host cell membrane trafficking pathways (Schroeder et al., 2011). Our study found that fungal EPs could enhance the phagocytic-killing activity of macrophages against STM infection in a concentration-dependent manner. Interestingly, all selected three fungal EPs at a concentration of 50 µg/mL significantly reduced the number of *Salmonella* present within macrophage cells. Notably, pretreatment of SC-EPs at a concentration of 25 µg/mL specifically demonstrated the reduction of the recovered number of intracellular STM. Cheng et al. (2019) also reported that pretreatment of the polysaccharide fraction extracted from the wild *Lactarius deliciosus* at least 25 µg/mL significantly enhanced the phagocytosis activity of RAW264.7. Moreover, polysaccharides from the mycelium of *Cordyceps sinensis* UM01 (15 µg/mL) were found to enhance the phagocytic activity of macrophages by 3.3-fold. This was evidenced by the increased uptake of FITC-dextran by RAW264.7 macrophages compared to the untreated control group (Cheong et al., 2016). The fungal EPs significantly promote the phagocytic function of macrophage cells by enhancing phagocytic uptake, which is important for boosting the immunomodulatory potential. This study focused on the phagocytic-killing activity of macrophages in response to STM. It involves subjecting macrophages to a preliminary treatment with fungal EPs, known for their immunomodulatory capabilities, followed by deliberate infection with STM. Thus, fungal EPs can improve the functioning and killing activity of RAW264.7, resulting in reducing the number of STM in intracellular macrophages during phagocytosis activity. These findings suggest that fungal EPs from mycelial culture broth have an immunomodulatory effect on the macrophage in STM infection.

Several studies have determined the inflammatory cytokines and inducible nitric oxide synthase (iNOS) production of RAW264.7 cells in cell culture supernatants by enzyme-linked immunosorbent assay (ELISA) and Western blot (Xiang et al., 2015; Zhang et al., 2022). Macrophages can be stimulated by several factors, such as pathogen-associated molecular patterns (PAMPs), including bacterial lipopolysaccharide (LPS), peptidoglycan, and lipoteichoic acid, as well as various pro-inflammatory cytokines. One of the most extensively studied PAMPs is bacterial LPS, which is found in the outer membrane of Gram-negative bacteria such as *Salmonella* (Rutledge et al., 2011; Wang et al., 2019a; Yamamoto et al., 2020; Chen et al., 2023b). Recognition of LPS occurs through the Toll-like receptor 4 and myeloid differentiation factor 2 complex (TLR4-MD2) mainly on macrophages (Park and Lee, 2013). When stimulated with LPS, macrophages show increased production of nitric oxide (NO) and elevated expression levels of tumor necrosis factor (TNF)-*α*, interleukin (IL)-1*β*, and IL-6 (Facchin et al., 2022). According to our findings, the production of pro-inflammatory and chemo-attractant cytokines was assessed by the expression of *Il6*, *Mip2*, *Nos2*, and *Tnfα* using RT-qPCR. Pretreatment of the RAW264.7 cells with fungal EPs significantly increased the upregulation of these pro-inflammatory genes in a dose-dependent manner. Our results suggest that these three fungal EPs can stimulate the innate immune response against STM infection. Additionally, specific assays (such as the Limulus amebocyte lysate (LAL) assay or ELISA for LPS detection) should be incorporated to confirm the absence of LPS or any other endotoxin contamination in the EPs derived from different fungal strains, ensuring that the observed macrophage stimulation is attributable to the fungal EPs (Domingues et al., 2012; He et al., 2019; Li et al., 2020).

Consistent with fungal EPs from various fungal species, they have been shown to activate macrophages. For example, EPs from *Paecilomyces lilacinus* PH0016 have been found to possess immunomodulatory properties on RAW264.7 cells. Treatment with these fungal EPs resulted in increased expression of inducible nitric oxide synthase (iNOS) and secretion of interleukin (IL)-1*β*, tumor necrosis factor (TNF)-*α*, and nitric oxide (NO) in macrophages (He et al., 2019). Similarly, EPs from culture broth *Trichoderma pseudokoningii* stimulate the activation of macrophages and induces the production of NO, IL-1β, and TNF-α (Wang et al., 2016). Purified polysaccharides from *Cordyceps cicadae* also exhibit good immunomodulatory activity on RAW264.7 macrophages in a concentration range of 50–400 µg/mL by enhancing the iNOS production and the secretion of the major inflammatory cytokines (IL-1*β*, IL-6, IL-12, and TNF-*α*) (Gao et al., 2023). Moreover, fungal EPs isolated from the culture broth of *Cordyceps cicadae*, *Aspergillus aculeatus*, *A*. *terreus*, and *Trichoderma* sp. KK19L1 showed a potent immunomodulatory effect by enhancing the production of NO, TNF-*α*, and IL-6 in a concentration-dependent manner from RAW264.7 (Li et al., 2020; Yang et al., 2023). Furthermore, some fungal EPs not only enhance the immunomodulatory functions of innate immunity but also boost the adaptive immune response. EPs from the culture supernatant of *Phellinus linteus* mycelium induced effective production of TNF-*α*, monocyte chemo-attractant protein (MCP)-1, IL-6, and NO in RAW264.7 and stimulated the Th1-cytokine interferon (IFN)-*γ* production in splenocytes (Shin et al., 2022). The mRNA expression levels of several cytokines (TNF-*α*, IFN-*γ*, and IL-2) in splenocytes and thymocytes were increased after the treatment with EPs from the cultured supernatant of *Cordyceps sinensis* (Sheng et al., 2011). However, further research on the effects of fungal EPs on anti-inflammatory cytokines (e.g., IL-10, and transforming growth factor-beta; TGF-*β*) in macrophages will be considered to provide a more comprehensive understanding of their immunomodulatory properties. Several previous studies have reported that crude EPs, isolated from liquid cultures via ethanol precipitation, can effectively activate and modulate immune responses. These findings support the potential use of crude fungal EPs as immunostimulants for future biomedical applications (Li et al., 2018; Ghosh et al., 2019, 2021; Santra and Banerjee, 2022; Zhong et al., 2023). However, ethanol precipitation may also co-precipitate fungal cell wall components such as chitin, *α*- and *β*-glucans, and glycoprotein contaminants (Złotko et al., 2019; Garcia-Rubio et al., 2020; Osibe et al., 2020; Fornal et al., 2023), which themselves possess immunomodulatory properties. Therefore, to confirm that the observed immunostimulatory effects are truly attributable to fungal EPs, future studies should incorporate specific immunoassays, such as ELISA, immunofluorescence, and *β*-D-glucan assays, to detect potential cell wall contaminants in crude EP preparations. Moreover, the use of purification techniques including dialysis, deproteination, enzymatic degradation with polysaccharide-degrading enzymes (e.g., endo-*β*-glucanase, chitinase), ion-exchange chromatography, and size-exclusion chromatography can help determine whether the observed immunostimulatory effects are truly attributable to the fungal EPs themselves rather than residual fungal cell wall components (Zheng et al., 2014; Sun et al., 2022; Fornal et al., 2023; Yang et al., 2023).

Besides, the RT-qPCR results of our study showed the downregulation of iNOS and TNF-*α* mRNA expression in STM-infected RAW264.7 cells when the concentration of SC-EPs was increased from 50 µg/mL to 200 µg/mL for pretreatment. In agreement with a study by Wang et al. (2019b), pretreatment of EPs from *L*. *edodes* inhibited mRNA expression levels of iNOS and TNF-*α* in a concentration-effect manner. Similarly, the anti-inflammatory activity of EPs from *S*. *commune* was assessed to significantly inhibit iNOS expression levels in LPS-induced RAW264.7 cells at a higher concentration (Du et al., 2017). Moreover, treatment of *L. edodes* polysaccharide to RAW264.7 at high concentration decreased the secretion of NO, but the production of IL-6 remained stable (Xu et al., 2012). Our findings suggest that fungal EPs from *S. commune* may have anti-inflammatory activity on RAW264.7 when the expression of other inflammatory cytokines (IL-6 and MIP-2) is excessive.

Overall, RAW264.7 macrophages are involved in a series of intricate steps that play a vital role in the immune response against pathogens. Treatment of RAW264.7 cells with our fungal EPs stimulated the robust expression of pro-inflammatory and related cytokines (IL-6, TNF-*α*, MIP-2, and iNOS) in a dose-dependent manner through several pathways, mainly involving MAPKs and NF-κB signaling during macrophage activation. Activated macrophages can also secrete ROS and reactive nitrogen species (RNS) in response to pathogenic infection (Subbian et al., 2007). RNS are a family of antimicrobial molecules derived from NO and superoxide produced via the enzymatic activity of iNOS and NADPH oxidase. Fungal EPs activate macrophages and up-regulate the expression of iNOS leading to enhanced levels of NO. The production of NO and TNF-*α* is an important part of the cytotoxic activity of macrophages. NO is recognized as an intercellular messenger and participates in signal transduction for immune regulation (Wu et al., 2013). TNF-α can induce the expression of inflammatory cytokines and other mediators (Glucksam-Galnoy et al., 2013). Likewise, MIP-2 and IL-6 assist in the recruitment of immune cells to the infection site, thereby modulating immune and inflammatory responses (Qin et al., 2017; Coperchini et al., 2021). When *Salmonella* infection occurs, PRRs/PAMPs interactions activate the phagocytic function of macrophages. This recognition triggers transduction signaling pathways, ultimately leading to the high production of inflammatory cytokines and mediators. Inflammatory responses are initiated upon exposure to certain cytokines, recruiting macrophages and other immune cells to the site of infection (Mogensen, 2009). These cytokines and inflammatory mediators serve as essential messengers both intracellularly and extracellularly for macrophages, playing a crucial role in immune defense against pathogenic bacteria (Nworu et al., 2015; Liu et al., 2021). Activated macrophages treated with fungal EPs exhibit an elongated and spindle-shaped appearance, increasing the area of contact with outer substances, which aids in phagocytic uptake in a concentration-dependent manner (Cheng et al., 2019; Yang et al., 2023). This morphological change is associated with an enhanced phagocytosis activity of the macrophages, enabling them to engulf pathogenic bacteria. Following phagocytosis, macrophages activate enzymes and antimicrobial molecules to degrade and neutralize the bacteria. Additionally, fungal EPs-treated macrophages may boost the production of ROS, NO, and several related cytokines (Hirayama et al., 2017). The heightened production of ROS and RNS plays a crucial role in effectively killing phagocytosed bacteria (Iovine et al., 2008; Didier et al., 2010). Furthermore, macrophages treated with fungal EPs not only produce pro-inflammatory cytokines, RNS, and ROS, but they also generate the production of cellular acid phosphatase (ACP) and lysozyme (Yang et al., 2023). ACP is a marker enzyme that participates in immune regulation, enhances the rates of phagocytosis, and aids in pathogenic clearance (Chen et al., 2021). Additionally, lysozyme can hydrolyze bacterial cell wall peptidoglycan, leading to bacterial cell lysis and death (Ragland and Criss, 2017). Taken together in our present study, all these related and complex mechanisms promote the full activation of macrophages ready for bacterial infection defense, resulting in the inhibition of intracellular survival and reduction of the number of *Salmonella* recovered within macrophages. These properties of fungal EPs could achieve the ability to limit the survival of pathogenic bacteria in intracellular macrophages. Investigating the interplay between *Salmonella* infection and the inflammatory cytokine production of fungal EPs-treated RAW264.7 macrophages offers insights into immunomodulation mechanisms and unveils potentially synergistic implications for defending against bacterial infection, especially for MDR STM.

## 5 Conclusion

The type of liquid media, initial pH value, incubation time, and fungal strain all influence the attainment of maximum EP yield. The findings in this study showed that *S. commune*, *G. fornicatum*, *G. williamsianum*, *E. scabrosa*, *F. tenuiculus*, and *P. sanguineus* produced the maximum EP yield in PDB. However, the maximum EP yield from *L*. *sajor-caju* was obtained in MCMB. Remarkably, this is the first report for EP production from the genera *Earliella* and *Favolus*. The slightly alkaline conditions of the liquid culture media could enhance EP production. The highest EP yield from the selected fungi was obtained after 10 to 14 days, depending on the fungal strain. The FT-IR spectroscopy analysis detected the major characteristics and functional groups of the crude fungal EPs, including hydroxyl, methyl, methylene, and carbonyl groups, as well as glycosidic bonds. This confirmed the typical carbohydrate patterns. Additionally, the crude fungal EPs were primarily comprised of glucose, followed by fructose, allose, and allulose, with differences based on the fungal strain. All crude fungal EPs demonstrated positive antioxidant potential in both the ABTS and DPPH assays. The immunomodulatory effects of selected crude EPs from *S. commune*, *G. fornicatum*, and *L. sajor-caju* against *Salmonella* infection were investigated. These EPs showed no cytotoxicity on cell viability and increased the proliferation of RAW264.7 macrophages at concentrations ranging from 25 to 1,000 μg/mL. Interestingly, the fungal EPs could enhance the phagocytic-killing activity of macrophages to inhibit the intracellular survival of STM. In addition, fungal EPs-pretreated RAW264.7 cells significantly upregulated the mRNA expression levels of several pro-inflammatory, and chemo-attractant cytokines in STM-infected macrophages. The results suggest that these three fungal EPs have a potential to be an alternative to antibiotics for STM infection by the modulation of host innate immune response. However, further *in vivo* studies for anti-*Salmonella* and immunomodulatory effects of these three fungal EPs are essential to aid in developing potential applications of fungal EPs in food, therapeutics, and pharmaceuticals.

## Data Availability

The original contributions presented in the study are included in the article/supplementary material. Further inquiries can be directed to the corresponding authors.
